# Vanillin Beyond Flavor: Therapeutic Potentials and Emerging Applications in Hydrogel-Based Biomaterials

**DOI:** 10.3390/gels12010016

**Published:** 2025-12-24

**Authors:** Lei Cui, Dong Uk Yang, Jing Liu, Ramya Mathiyalagan, Jong-Hoon Kim, Sathiyamoorthy Subramaniyam, Changbao Chen, Deok-Chun Yang, Ling Li

**Affiliations:** 1 Jilin Ginseng Academy, Changchun University of Chinese Medicine, Changchun 130117, China; cuilei@ccucm.edu.cn (L.C.); 270000138@ccucm.edu.cn (J.L.);; 2 Mongsim Corporation, Daejeon 34916, Republic of Korea; dongukyang83@gmail.com; 3 Graduate School of Biotechnology, College of Life Science, Kyung Hee University, Yongin-si 17104, Republic of Korea; 4 Department of Biotechnology, College of Fisheries Sciences, Pukyong National University, Busan 48547, Republic of Korea; 5 Department of Marine Biotechnology, AMET University, Chennai 603112, Tamil Nadu, India

**Keywords:** vanillin, pharmacological properties, hydrogels, biomaterials, wound healing, drug delivery, tissue engineering

## Abstract

Vanillin (4-hydroxy-3-methoxybenzaldehyde) is widely recognized for its aromatic flavor and established pharmacological properties, including antioxidant, antimicrobial, anti-inflammatory, and anticancer effects. While these biological activities underpin its therapeutic potential, recent advances have expanded the application of vanillin into the field of biomaterials. In particular, vanillin’s unique chemical structure enables its use as a multifunctional building block for the development of innovative hydrogels with dynamic covalent bonding, injectability, and self-healing capabilities. Vanillin-based hydrogels have demonstrated promising applications in wound healing, drug delivery, tissue engineering, and antimicrobial platforms, combining structural support with intrinsic bioactivity. These hydrogels benefit from vanillin’s biocompatibility and functional versatility, enhancing mechanical properties and therapeutic efficacy. This review provides an overview of vanillin’s pharmacological effects, with a primary focus on the synthesis, properties, and biomedical applications of vanillin-derived hydrogels. By highlighting recent material innovations and their translational potential, we aim to position vanillin as a valuable natural compound bridging bioactivity and biomaterial science for future clinical and therapeutic advancements.

## 1. Introduction

**Figure 1:**
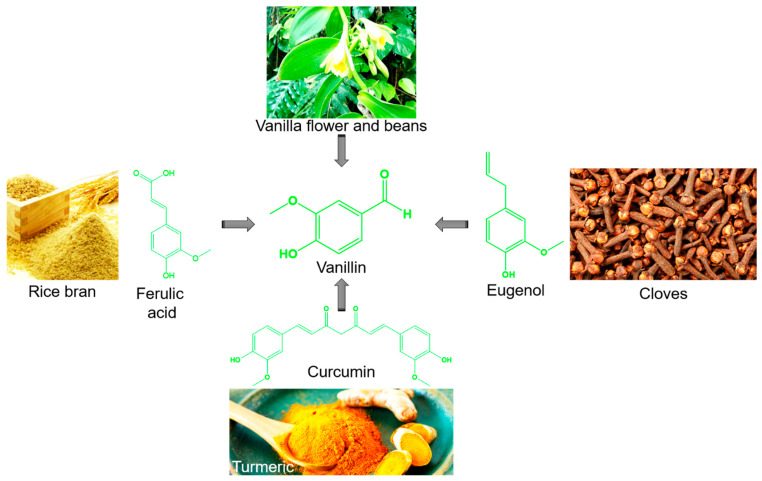
Various natural sources and production pathways of vanillin.

Vanillin, a 4-hydroxy-3-methoxybenzaldehyde, is the primary flavor compound responsible for the characteristic aroma of vanilla [1]. It can be produced from various sources and methods. Vanillin is naturally extracted from vanilla beans of the *Vanilla planifolia* orchid, though this process is labor-intensive and costly [2]. Most vanillin used today is synthesized either chemically or biologically. Synthetic vanillin is commonly derived from guaiacol or lignin, a byproduct of the paper and pulp industry, offering a sustainable and economical alternative [3]. Biotechnological advancements also allow vanillin production through microbial fermentation, using substrates like ferulic acid from plant waste [4,5,6]. These diverse production methods cater to growing demand in food, fragrance, and pharmaceutical industries, highlighting vanillin’s versatility and sustainability. Various natural sources and production pathways of vanillin are shown in Figure 1. Vanillin can be obtained from natural sources or produced synthetically and biotechnologically, with the latter routes now dominating due to scalability, cost efficiency, and sustainability.

Vanillin, a phenolic aldehyde with the chemical formula 4-hydroxy-3-methoxybenzaldehyde, is most recognized for its characteristic sweet, creamy aroma, which is the primary flavor component of vanilla [7]. It is moderately soluble in water, but more readily dissolves in alcohol and other organic solvents, making it ideal for use in various food, beverage, and fragrance formulations. Beyond its sensory role, vanillin exhibits well-documented antioxidant, anti-inflammatory, antimicrobial, anticancer, and neuroprotective activities, positioning it as a multifunctional bioactive compound for biomedical applications [8,9,10,11]. While generally stable, prolonged exposure to heat or light can cause vanillin to degrade, altering its aroma and effectiveness. These versatile properties make vanillin valuable across multiple industries, including food, cosmetics, and pharmaceuticals.

Due to emerging reports highlighting vanillin’s potential as a therapeutic molecule and its inclusion in the “Generally Recognized As Safe” (GRAS) list for food additives, it has gained attention as a promising candidate for healthcare applications [12]. In this review, we aim to provide a comprehensive examination of vanillin’s bioactive properties, exploring its potential to be recognized as a mainstream bioactive small molecule. We discuss its various pharmacological activities and the screening of vanillin in a range of in vitro and in vivo models, highlighting its mechanistic effects. Through this analysis, we explore the therapeutic potential of vanillin and its applications across multiple health-related domains, paving the way for its broader integration into medical and pharmaceutical practices. Future directions for vanillin research focus on further exploring its therapeutic potential through clinical trials to validate its efficacy and safety in diverse medical applications. Additionally, developing advanced formulations and delivery systems to enhance its bioavailability and targeted action could expand its use in personalized medicine.

Beyond its pharmacological effects, vanillin’s functional groups and aromatic structure have also attracted increasing attention in the field of biomaterials. The phenolic hydroxyl and aldehyde groups in vanillin enable facile chemical modification and make it an excellent candidate for use as a cross-linking agent or functional additive in polymer networks. These reactive sites allow vanillin to participate in Schiff base formation with amine-containing polymers, Michael addition reactions, and other dynamic covalent interactionskey mechanisms in hydrogel formation. As a result, vanillin has been incorporated into a variety of hydrogel systems, especially those based on natural polymers such as chitosan, gelatin, alginate, and hyaluronic acid.

Vanillin-based hydrogels have demonstrated unique characteristics such as injectability, self-healing ability, and pH-responsiveness, driven by dynamic covalent bonds that enable reversible network formation. These features make vanillin-derived hydrogels especially suitable for biomedical applications where both structural integrity and biological functionality are critical.

Moreover, vanillin has been utilized to enhance the biological interface of synthetic biomaterials, enabling better cellular adhesion, proliferation, and immune compatibility. The biodegradability and tunability of vanillin-derived hydrogels offer additional advantages for applications in tissue engineering scaffolds and localized drug delivery platforms. Recent advances also include the integration of vanillin into 3D bioprinting inks, allowing the fabrication of spatially organized, bioactive structures for regenerative medicine. Despite extensive studies on vanillin’s pharmacological effects and growing reports on vanillin-functionalized hydrogels, a consolidated analysis linking vanillin’s chemical functionality to hydrogel structure, properties, and biomedical performance remains lacking.

In this review, we provide an overview of recent developments in vanillin-based hydrogel design, fabrication strategies, physicochemical properties, and biomedical applications. By bridging the domains of small-molecule therapeutics and macromolecular biomaterials, vanillin represents a unique, multifunctional building block that offers exciting opportunities for future translational research in health and materials science. To strengthen the scientific basis of vanillin-based biomaterials, this review critically evaluates recent experimental evidence, elucidates underlying molecular mechanisms, and highlights structure–property relationships governing vanillin-functionalized hydrogel systems. Beyond summarizing existing reports, we integrate comparative analyses and identify emerging biomedical opportunities that have not been comprehensively reviewed previously. The novelty of this review lies in synthesizing recent mechanistic insights, analytical comparisons, and translational perspectives on vanillin-functionalized hydrogels, offering a research-centered framework beyond descriptive summaries presented in earlier literature.

To guide the reader, this review is structured into major sections covering: (i) vanillin’s pharmacological and biological properties, (ii) strategies to improve vanillin’s therapeutic performance using biomaterials, (iii) the design, structural features, and biomedical applications of vanillin-based hydrogels, (iv) rheological and mechanical considerations of vanillin-functionalized hydrogels, and (v) current challenges, limitations, and future research directions.

## 2. Pharmacological Properties of Vanillin

Vanillin, the primary component of vanilla extract, possesses a wide range of pharmacological properties, making it valuable in therapeutic and nutraceutical applications [12]. It exhibits strong antioxidant and anti-inflammatory activities, protecting against oxidative stress and inflammation-related diseases. Its antimicrobial properties are effective against bacteria, fungi, and biofilm formation, while its anticancer potential involves inducing apoptosis and inhibiting tumor growth. Vanillin also demonstrates neuroprotective effects, cardioprotective benefits by improving vascular health, and anti-diabetic properties through enhanced glucose metabolism and insulin sensitivity. Additionally, it serves as an analgesic, promotes wound healing by supporting collagen synthesis, and protects the liver and gastric lining from damage [13,14]. These attributes highlight vanillin’s versatility in addressing various health conditions.

### 2.1. Anticancer Properties

Considerable evidence highlights vanillin’s potential in cancer treatment and prevention across various models. Research emphasizing vanillin’s capacity to mediate DNA damage and its antimutagenic properties has sparked significant interest among scientists. These findings have encouraged further exploration of vanillin’s anticancer potential, focusing on its mechanisms of action at both cellular and molecular levels to better understand its therapeutic applications. Early studies demonstrated its antimutagenic effects, as vanillin reduced mutation rates caused by mitomycin C and methylmethane in mouse bone marrow cells and *Drosophila melanogaster* [15]. Vanillin also countered genotoxicity by inhibiting chromosome-related aberrations and shielding against mutational processes. Furthermore, vanillin demonstrated its ability to suppress mutations induced by mutagenic agents such as N-ethyl-N-nitrosourea and bleomycin in the somatic cells of *Drosophila melanogaster*, highlighting its potential as an antimutagenic compound in genetic model systems [16].

In colon cancer studies, vanillin, at a concentration of 1000 μg/mL, effectively inhibited the proliferation of HT-29 cells by inducing cell cycle arrest during the G0/G1 phase and increased apoptotic cells in the sub-G0 phase [17]. A vanillin derivative, 4-(1H-imidazo [4,5-f][1,10]phenanthrolin-2-yl)-2-methoxyphenol (IPM711), exhibited significant anticancer activity by inhibiting the growth of HT-29 and HCT-116 colon cancer cells [18]. It also effectively suppressed cell invasion and migration, primarily by targeting receptors involved in the Wnt/β-catenin signaling pathway, a critical regulator of cancer progression and metastasis. Vanillin was found to have down-regulated proteasome-related genes in colon tissues, suppressed proteasome activity, and inhibited MAPK phosphorylation at 10 mM, reducing granulocytes, proliferating cells, and p65-positive cells in colon tissues. These effects suggest that vanillin’s anticancer activity in colon tissues is associated with the down-regulation of proteasome genes, the mitogen-activated protein kinases (MAPKs) pathway, and nuclear factor-κB.

**Figure 2:**
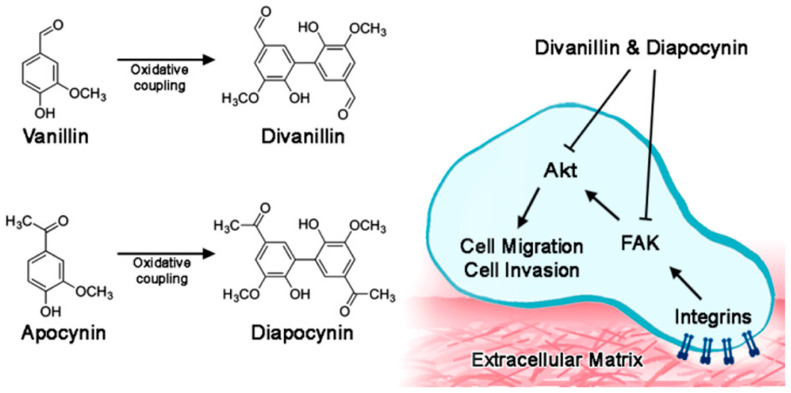
The monodimer of vanillin inhibits focal adhesion kinase (FAK) using FAK inhibitor I and phosphoinositide 3-kinase (PI3K) using LY294002, thereby blocking the activation of both FAK and the PI3K/Akt signaling pathways, which are crucial for cell migration, survival, and metastasis. Reprinted with permission from Ref. [20]. 2017, American Chemical Society Publications.

Vanillin’s anticancer properties extend to the regulation of metastasis. In human non-small cell lung carcinoma cells (NCI-H460), vanillin effectively inhibited metastatic potential by down-regulating the expression of caveolin-1 (Cav-1), a protein associated with tumor progression. Additionally, in HepG2 liver cancer cells, vanillin reduced the gelatinolytic activity of matrix metalloproteinase-9 (MMP-9) by suppressing the nuclear factor kappa B (NF-κB) signaling cascade, highlighting its multifaceted role in curbing cancer spread [19]. Furthermore, a monodimer of vanillin demonstrated the ability to reduce the metastatic potential of HepG2 liver cancer cells by suppressing the focal adhesion kinase (FAK)/PI3K/Akt signaling pathway, a critical regulator of cell migration, invasion, and survival in cancer progression (Figure 2) [20]. In addition to its effects on colon and liver cancer, vanillin has been shown to induce apoptosis in neuroblastoma cells and human hepatic carcinoma, highlighting its potential as a therapeutic agent capable of triggering programmed cell death in diverse types of cancer. Molecular docking studies indicate that vanillin has a strong binding affinity for calcium/calmodulin-dependent protein kinase IV (CaMKIV), an enzyme implicated in the progression of cancer and neurodegenerative diseases [21]. This interaction suggests a potential mechanism through which vanillin exerts its therapeutic effects.

In in vivo studies, inclusion of 1% vanillin in the diet reduced tumor incidence and the number of tumors in a rat multi-organ carcinogenesis model [22]. In mice, vanillin administered at doses ranging from 125 to 500 mg/kg effectively suppressed ethylnitrosourea-induced mutations, induced apoptosis, and enhanced antioxidant activity in Ehrlich tumor-bearing mice, demonstrating its potential as a therapeutic agent for both mutagenesis and tumor growth inhibition. When combined with doxorubicin, it further improved the Bax ratio, activated caspase-9, and inhibited breast cancer cell (MCF-7) growth, suggesting its utility as a standalone or adjunct therapy [23]. The vanillin derivative VND3207 also demonstrated a strong radioprotective effect. It alleviated radiation-induced intestinal injury in mice and protected human lymphoblastoid cells. This protective effect was attributed to the enhancement of DNA-dependent protein kinase (DNA-PKcs) expression, a key component in DNA double-strand break repair mechanisms [24]. Vanillin also mitigated radiation-induced DNA damage, protecting human leukocytes and mouse blood cells from γ-radiation through its antioxidant properties and DNA repair mechanisms [25]. Vanillin’s ability to quench free radicals, inhibit NF-κB cascades, and suppress tumor genes has been demonstrated across various models of breast, liver, colon, and lung cancers.

While the findings are promising, variability in experimental conditions complicates identifying the most effective application. Multi-omics and modeling approach could help a precise identification of vanillin’s molecular targets. Robust, drug discovery-oriented research is essential to establish its role as an essential component of anticancer therapies or functional foods.

### 2.2. Antimicrobial Properties

Studies have demonstrated that vanillin exhibits significant antimicrobial activity against various microorganisms, including bacteria, molds, and yeasts [9]. For instance, vanillin demonstrated minimum inhibitory concentration (MIC) values of 15 mM against *E. coli*, 75 mM against *Lactobacillus plantarum*, and 35 mM against *Listeria innocua*, highlighting its antimicrobial potential against various bacterial strains [26]. Vanillin also demonstrated an inhibitory effect on the growth of spoilage bacteria, including *Pantoea agglomerans*, *Aeromonas enteropelogenes*, *Micrococcus lylae*, and *Sphingobacterium spiritovorun*, with minimum inhibitory concentrations (MIC) ranging from 10 to 13.3 mM. Additionally, exposure to 10–40 mM vanillin was found to inhibit respiration in *E. coli* and *L. innocua*. At concentrations of 50–100 mM, vanillin caused the complete dissipation of proton ion gradients and the loss of pH homeostasis in *Lactobacillus plantarum*, further indicating its bactericidal potential. In proteomic studies of *E. coli* treated with vanillin, around 147 proteins showed remarkable changes in abundance in response to the compound. This treatment resulted in the accumulation of reactive oxygen species (ROS), which triggered adaptive responses mediated by the MarA, OxyR, and SoxS regulatory network. Additionally, there was an increase in gene expression dependent on the RpoS/DksA pathway. Furthermore, AcrD and AaeAB were identified as potential efflux systems responsible for vanillin resistance, suggesting their role in mitigating the toxic effects of vanillin on bacterial cells. These findings suggest that vanillin’s antimicrobial mechanisms may involve complex cellular responses, and further omics-based studies are needed to explore potential gene/protein targets in other pathogenic bacteria, particularly those identified as critical threats by the World Health Organization.

**Figure 3:**
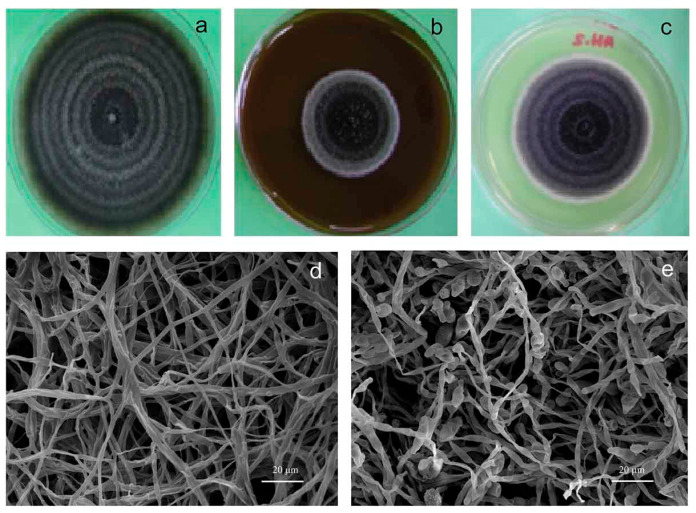
Effects of vanilla juice and commercial-grade vanillin (250 mg/L) on A. alternata: Control (**a**), vanilla juice (**b**), commercial-grade vanillin (**c**), hyphal effects (**d**), and normal conidia and hyphae morphology (**e**). Reprinted with permission from Ref. [29]. 2019, Taylor & Francis Online.

Regarding antifungal properties, Fitzgerald et al. reported that vanillin exhibited antifungal activity against yeasts and molds commonly associated with food spoilage, with an average minimum inhibitory concentration (MIC) value of 5.71 mM, further supporting its potential as a natural preservative [27]. Vanillin effectively inhibits growth of these fungi in soft drinks and agar systems containing purees and fruits, suggesting its potential as an anti-spoilage agent [28]. Further studies revealed that vanillin inhibits the growth of medically important fungi, including *Cryptococcus neoformans* (MIC: 293 μg/mL; MFC: 521 μg/mL) and *Candida albicans* (MIC: 625 μg/mL; MFC: 1209 μg/mL), an effect attributed to its aldehyde group [27]. Additionally, vanillin isolated using *Vanilla planifolia* pods exhibited antifungal activity against *Alternaria alternata*, reducing its growth at a concentration of 750 mg/L (Figure 3) [29]. These findings highlight vanillin’s dual antibacterial and antifungal properties, making it a valuable agent for preventing food and drink spoilage. Consequently, vanillin is widely used in sweetened foods, such as cookies, muffins, and soft drinks, to extend their shelf life while helping to preserve their taste and appearance, making it a valuable ingredient in the food industry for maintaining product quality.

Some derivatives of vanillin have also demonstrated potent antimicrobial properties against pathogenic microbes. Numerous Schiff bases derived from vanillin demonstrated inhibitory properties against both Gram-negative and Gram-positive bacteria, including *Staphylococcus subfava*, *Citrobacter freundii*, *Bacillus megaterium*, *Pseudomonas pseudoalcaligenes*, *Enterobacter aerogenes*, and *Proteus vulgaris*. Additionally, Sridevi et al. reported that Schiff bases (SB-1 to SB-6) synthesized from vanillin exhibited promising antibacterial activity against a range of bacterial strains, including Gram-positive bacteria such as *Staphylococcus aureus* and *Bacillus subtilis*, as well as Gram-negative bacteria like *Klebsiella pneumoniae* and *Pseudomonas aeruginosa*, suggesting their potential as effective antimicrobial agents [7]. Among these Schiff bases, SB-5 and SB-6 exhibited the most promising antimicrobial activity, with minimum inhibitory concentration (MIC) values of 125 μg/mL against both Gram-positive and Gram-negative bacteria, demonstrating their broad-spectrum potential.

These findings indicate that vanillin serves not only as an antimicrobial agent but also as a precursor for developing derivatives with enhanced antibacterial properties. Notably, while vanillin exhibits antimicrobial effects against bacteria, its antifungal activity tends to be more potent, underscoring its diverse applications in food preservation and potential therapeutic developments.

### 2.3. Antioxidant and Anti-Inflammatory Properties

Available information reveals that vanillin possesses significant antioxidant activity. In in vitro models, vanillin scavenged several types of free radicals, such as 1,1-diphenyl-2-picrylhydrazyl (DPPH) (IC_50_: 0.61 μM), hydroxyl radicals (IC_50_: 0.16 μM), and superoxide anion radicals (IC_50_ values: 2.95 mM and 2.33 μM), underscoring its potential as a potent natural antioxidant [30]. It also inhibited lipid peroxidation, exhibiting IC_50_ values of 3.29 mM and 0.37 μM. Besides inhibiting lipid peroxidation, vanillin slowed down protein peroxidation induced by photosensitization in the mitochondria of rat liver. The compound forms an adduct with radicals, exhibiting strong ROS quenching ability, and it also inhibited the excessive production of advanced glycation end products.

Further studies in in vivo models have indicated vanillin’s antioxidant action. Kadeche et al. reported that vanillin exhibited ameliorative effects in response to metribuzin-induced oxidative stress in rats, suggesting its potential as a protective agent against chemically induced oxidative damage [31]. In this research, vanillin administered at a dose of 150 mg/kg effectively reduced the damage caused by metribuzin exposure. It did so by lowering the levels of malondialdehyde, a marker of oxidative stress, and enhancing the activity of antioxidant defense enzymes, which help protect cells from oxidative damage. Additional in vivo studies have corroborated these findings, further supporting vanillin’s potential as an agent to counteract oxidative stress and damage induced by toxic substances like metribuzin. Intraperitoneal administration of vanillin at a dose of 100 mg/kg in mice models with potassium bromate-induced oxidative damage led to a significant reduction in protein and lipid peroxidation levels. Additionally, vanillin treatment resulted in an improvement in the activity of antioxidant enzymes, which are crucial for neutralizing oxidative stress and protecting cells from damage caused by reactive oxygen species. This suggests vanillin’s potential as a therapeutic agent in mitigating oxidative damage in vivo [32]. Additionally, vanillin administered at a dose of 150 mg/kg effectively reduced oxidative damage, specifically protein carbonylation, in rats exposed to carbon tetrachloride (CCl4), a known inducer of liver toxicity [33]. Vanillin also prevented erythrocyte osmotic fragility, which is a sign of cell membrane damage, and modulated the activities of crucial enzymes, such as Ca^2+^-ATPase and Na^+^/K^+^-ATPase. This treatment also preserved cell membrane integrity by preventing oxidative damage to mitochondrial membranes in rat liver cells. These findings collectively highlight vanillin’s curative effects against toxic oxidative damage and suggest its potential in managing oxidative stress-related disorders. Further research, especially human-based studies, is warranted to explore its broader applications.

**Figure 4:**
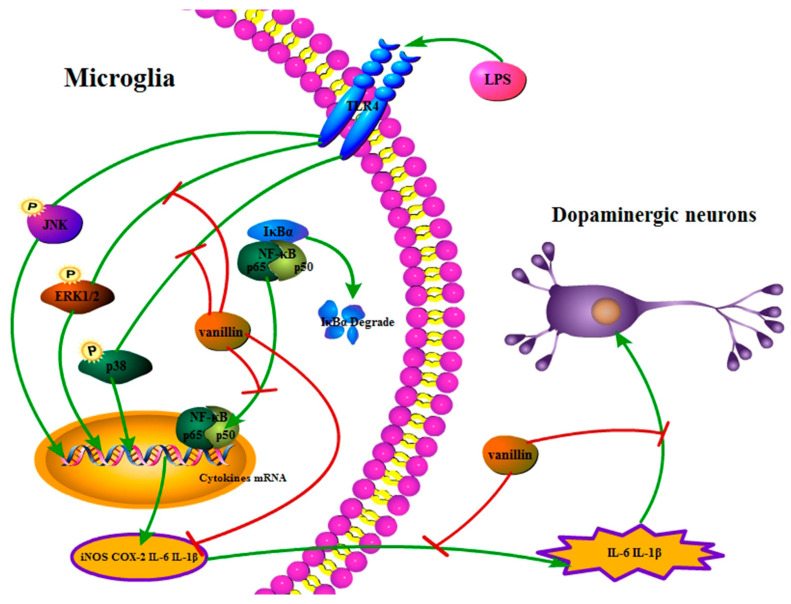
Vanillin protects dopaminergic neurons from inflammation-induced cell death by inhibiting ERK1/2, p38, and NF-κB signaling pathways. Reprinted with permission from Ref. [34]. 2017, Multidisciplinary Digital Publishing Institute.

Vanillin also displays anti-inflammatory and analgesic effects. Vanillin inhibited the activation of NF-κB triggered by lipopolysaccharide (LPS), downregulated cyclooxygenase-2 (COX-2) expression, and decreased the production of pro-inflammatory markers, including nitric oxide (NO) and inducible nitric oxide synthase (iNOS) in an in vivo model (Figure 4) [34]. Additionally, vanillin inhibited MAPKs in LPS-induced microglial cells [35]. In vivo studies have supported these findings. When C57BL/6 mice inhaled vanillin for 20 min, pain was suppressed in the hot plate test, without influencing other behavioral tests [36]. This suggests that inhalation of vanillin may possess antinociceptive (pain-relieving) and muscle relaxant properties, potentially making it useful for managing pain and muscle tension. These effects highlight vanillin’s therapeutic potential in alleviating discomfort related to muscle spasms or pain.

While these studies demonstrate vanillin’s anti-inflammatory actions, additional studies, such as membrane stabilization assays and protein denaturation, are needed to further validate its therapeutic potential in managing inflammation and related conditions.

### 2.4. Neuroprotective Properties

**Figure 5:**
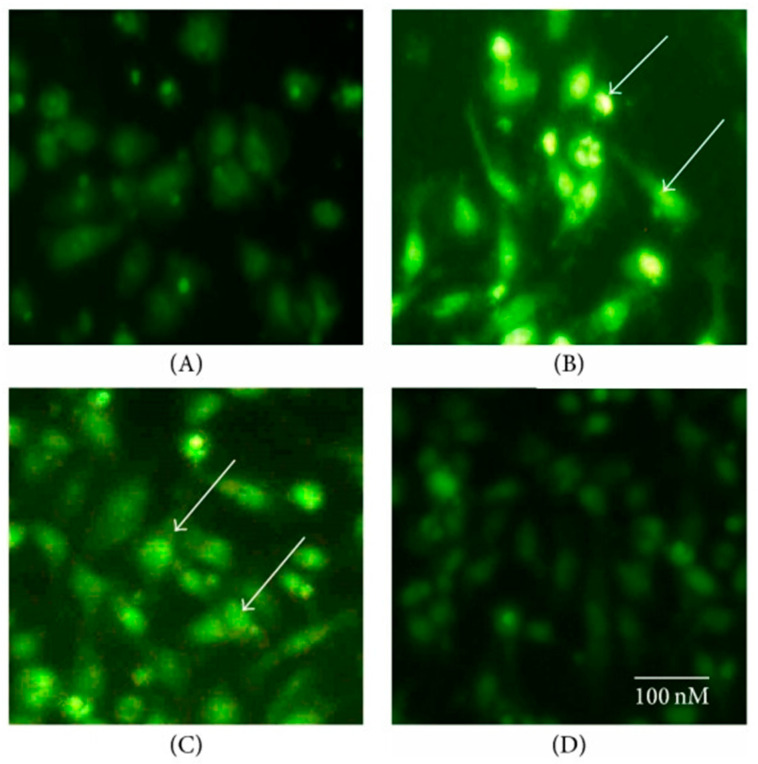
Vanillin (100 nM) reduced ROS formation, as visualized using 1 μM CM-H2 DCFDA staining. Photomicrographs show (**A**) Control, (**B**) Rotenone, (**C**) Vanillin + Rotenone, and (**D**) Vanillin. Reprinted with permission from Ref. [37]. 2015, Wiley Online library.

Numerous scientific reports have demonstrated the neuroprotective effects of vanillin. For example, in an in vitro study, pretreatment with vanillin protected SH-SY5Y neuroblastoma cells from rotenone-induced neurotoxicity by reducing apoptosis and mitochondrial dysfunction (Figure 5) [37]. This effect was achieved by upregulating c-Jun N-terminal kinase (JNK)-MAPK and p38 signaling pathways. The neuroprotective effect of vanillin was further confirmed in an in vivo rat model of Parkinson’s disease, where vanillin was administered at doses ranging from 5 to 20 mg/kg [38]. Treatment with vanillin resulted in significant improvements in neurochemical deficits, a reduction in apoptosis (cell death), and alleviation of both motor and non-motor impairments. Additionally, vanillin helped reduce oxidative stress, which is a key factor in the progression of neurodegenerative diseases like Parkinson’s, suggesting its potential as a therapeutic agent for managing Parkinson’s disease symptoms. Similarly, administration of vanillin at a dose of 150 mg/kg enhanced intramuscular vascularization in the tibialis anterior and soleus muscles of rats with nerve injury [39]. This suggests that vanillin may promote improved blood flow and tissue regeneration in injured muscles, which could support the recovery and healing process following nerve damage. Enhanced vascularization is crucial for delivering nutrients and oxygen to the injured tissues, aiding in their repair and function.

Additionally, vanillin demonstrated neuroprotective properties through the inhibition of butyrylcholinesterase and acetylcholinesterase activities, while also ameliorating oxidative damage in the brain tissues of rats subjected to Fe^2+^-induced toxicity [40]. In another report by Beaudry et al. suggested that vanillin at a dose of 50–100 mg/kg reduced allodynia in rats, without significantly affecting hyperalgesia, suggesting a potential in treating neuropathic pain and allodynia [41]. In conclusion, vanillin demonstrates neuroprotective effects by reducing apoptosis, relieving brain mitochondrial stress and dysfunction, improving the overall mitochondrial function that is often compromised during neurotoxic conditions. This action contributed to enhanced motor and non-motor impairments, suggesting that vanillin not only protects brain cells from oxidative damage but also supports the recovery of brain function, alleviating symptoms related to both physical movement and cognitive or emotional well-being. These results suggest that vanillin may play a beneficial role in managing neurological disorders, including neuropathic pain and Parkinson’s disease.

### 2.5. Hepatoprotective and Nephroprotective Properties

The hepatoprotective and nephroprotective properties of vanillin have been widely studied, highlighting its potential as a therapeutic agent for organ protection. Notably, vanillin has demonstrated anti-hepatotoxic effects in mice with potassium bromate-induced liver damage. It significantly reduced serum transaminases, markers of liver injury, as well as lipid peroxidation, protein carbonyls, and advanced oxidation protein products, all of which are indicators of oxidative damage. Additionally, vanillin improved liver architecture, suggesting its ability to promote liver regeneration and restore normal liver function following toxic damage. Similarly, intraperitoneal vanillin administration at a dosage of 150 mg/kg offered protection against CCl_4_-induced hepatotoxicity in rats [42]. This protective effect was evidenced by a decrease in serum transaminase activities, which are typically elevated in liver damage, indicating a reduction in liver injury. Additionally, there was a reduction in hepatic protein carbonyl synthesis and lipid peroxidation, both markers of oxidative stress and cellular damage. Vanillin also contributed to the improvement of liver architecture, suggesting tissue repair, and enhanced the oxidative defense system, further indicating its potential to protect the liver from damage and support its recovery.

Vanillin has also demonstrated nephroprotective effects. It was found to protect against cisplatin- and methotrexate-induced renal injury in rats [11,43]. This protective effect was associated with a decrease in serum creatinine, cystatin C, and neutrophil gelatinase-associated lipocalin, which are commonly elevated in kidney dysfunction and injury. Additionally, various kidney tissue biomarkers were reduced, including malondialdehyde (a marker of lipid peroxidation), iNOS (inducible nitric oxide synthase), TNF-α (tumor necrosis factor-alpha), IL-18 (interleukin-18), and NF-κB p65 (a key transcription factor involved in inflammation). The reduction in cytosolic cytochrome C and caspase-3 further indicated that vanillin helped prevent apoptosis and cellular damage in kidney tissues, suggesting its nephroprotective potential. Additionally, vanillin improved renal total antioxidant capacity and the Bcl-2/Bax ratio, while also mitigating histopathological injury and downregulating Fas ligand expression in the kidneys. At a lower dose (100 mg/kg), vanillin further alleviated kidney damage induced by cisplatin. This was supported by a reduction in fibrotic indices such as TGF-β1 (transforming growth factor-beta 1) and fibroblast growth factor-23 (FGF-23), both of which are involved in the development of fibrosis and kidney dysfunction. Additionally, vanillin treatment led to improvements in serum creatinine and urea levels, which are key indicators of kidney function. Overall, these findings suggest that vanillin not only mitigates oxidative and inflammatory damage but also helps preserve kidney integrity and function by reducing fibrosis and supporting renal recovery. Consequently, vanillin may serve as an effective agent in preventing liver and kidney damage caused by chemical insults such as cisplatin and potassium bromate.

### 2.6. Anti-Hyperglycemic and Anti-Hyperlipidemic Properties

Vanillin plays a crucial role in preventing and managing pathological conditions associated with hyperglycemia (high blood sugar) and hyperlipidemia (high blood lipid levels). Specifically, vanillin isolated from *Gastrodia elata* has been shown to reduce insulin resistance, a key factor in the development of type 2 diabetes, by decreasing fat accumulation in adipocytes (fat cells). It also stimulates lipolysis (the breakdown of fats), helping to reduce excess fat, and promotes leptin signaling, which regulates energy balance and appetite. These effects were observed in obese rats, indicating vanillin’s potential as a therapeutic agent for managing obesity-related metabolic disorders [44]. In a recent study, Lu et al. reported that vanillin administered at doses of 100 and 200 mg/kg body weight significantly reduced blood glucose levels by 54% and insulin levels in streptozotocin-induced diabetic neonatal rats [45]. Additionally, vanillin helped mitigate oxidative stress, a key factor in the development of diabetes and its complications. These findings suggest that vanillin may have potential as a therapeutic agent for managing blood glucose levels and oxidative damage in diabetic conditions. Moreover, vanillin improved several important biochemical markers in the diabetic rats, including serum levels of triacylglycerol and creatinine, as well as the activities of liver enzymes alanine aminotransferase (ALT) and aspartate aminotransferase (AST). These improvements suggest that vanillin may have protective effects on both metabolic and liver functions. Additionally, inflammatory markers such as TNF-α, monocyte chemoattractant protein-1 (MCP-1), interleukin-6 (IL-6), and interleukin-1β (IL-1β) were reduced, further indicating vanillin’s anti-inflammatory properties. The blood glucose-lowering effect of vanillin was attributed to its ability to reduce both inflammation and oxidative stress, highlighting its potential as a therapeutic agent for managing diabetes and its associated complications.

Additionally, vanillin (100 mg/kg body weight) was found to reduce fasting blood glucose by 28% and ameliorate diabetes-induced nephropathy [46]. This was achieved by downregulating proteinuria, advanced glycation end products (AGEs), and blood urea nitrogen levels. Vanillin also reduced malondialdehyde (MDA), IL-6, and transforming growth factor-β1 (TGF-β1) levels, along with the expression of NF-κB in kidneys. These findings suggest that vanillin’s antioxidant and anti-inflammatory properties contribute to its protective effect against diabetic nephropathy.

Furthermore, vanillin (200–400 mg/kg body weight) improved hyperlipidemia in mice fed a cholesterol-rich high-fat diet by lowering serum total cholesterol, triglycerides, and low-density lipoprotein cholesterol levels [47]. A recent study also demonstrated that vanillin, along with other structurally related compounds such as p-hydroxybenzaldehyde and syringaldehyde, exhibited antidiabetic properties. Notably, these effects were independent of the presence and positioning of methoxy groups on the aromatic ring, suggesting that the antidiabetic potential of these compounds may be attributed to their core molecular structure rather than specific modifications on the aromatic ring. This finding highlights the importance of the fundamental chemical structure in determining the bioactivity of these compounds, providing insights for the development of new antidiabetic agents. Therefore, vanillin shows potential as an effective agent for ameliorating hyperglycemia, hyperlipidemia, insulin resistance, and inflammation associated with diabetes. Although these pharmacological effects underscore vanillin’s therapeutic promise, their practical application is often constrained by issues such as poor solubility, rapid metabolism, and limited bioavailability, highlighting the need for material-based delivery and stabilization strategies.

## 3. Improving Pharmacological Properties of Vanillin Using Biomaterials

To overcome the intrinsic limitations associated with free vanillin administration, biomaterial-based approaches—particularly hydrogel systems—have emerged as effective platforms to enhance its stability, bioavailability, and therapeutic performance. Despite promising preclinical findings, challenges such as low bioavailability, rapid metabolism, and limited clinical validation hinder the broader therapeutic application of vanillin. Recent advancements in nanotechnology and biomaterials offer innovative solutions to overcome these limitations, significantly enhancing vanillin’s pharmacokinetic and therapeutic properties [48,49,50,51]. For instance, the encapsulation of vanillin in polymeric nanoparticles, such as polylactic acid nanoparticles, has been shown to improve its stability and prolong its release, ensuring sustained therapeutic action [52]. Studies have demonstrated that vanillin-loaded polylactic acid nanoparticles not only enhance its antioxidant and anticancer properties but also provide a controlled release profile, reducing dosing frequency and side effects.

Hydrogel-based delivery systems further exemplify the potential of biomaterials to improve vanillin’s pharmacological efficacy. Injectable hydrogels synthesized from natural polymers like chitosan, alginate, or hyaluronic acid can encapsulate vanillin, providing a localized and sustained release at the target site [53,54,55,56]. For example, chitosan-based hydrogels loaded with vanillin have shown enhanced wound healing properties by leveraging its antimicrobial and anti-inflammatory effects [57]. Vanillin incorporated into thermo-responsive hydrogels has demonstrated potential for anticancer therapy, as these hydrogels release the drug in response to the elevated temperatures of tumor microenvironments, ensuring localized action with minimal off-target effects.

Moreover, vanillin conjugation with nanoscale carriers such as liposomes, dendrimers, and micelles has been explored to improve solubility and bioavailability [58]. A notable example includes vanillin-loaded liposomes that exhibit enhanced neuroprotective effects by crossing the blood–brain barrier efficiently, a crucial factor for treating neurodegenerative diseases [59]. Additionally, vanillin complexed with cyclodextrins has demonstrated improved solubility and bioactivity [60], making it a promising approach for food preservation and therapeutic applications.

Vanillin exhibits concentration-dependent biological activity. In regenerative medicine, vanillin is generally used at low concentrations (10–200 µM), where it is well tolerated by normal cells such as fibroblasts, endothelial cells, and mesenchymal stem cells [10]. At these levels, vanillin often provides cytoprotective antioxidant effects rather than cytotoxicity [61]. In contrast, therapeutic anticancer activity typically occurs at higher concentrations (0.5–5 mM), where vanillin induces apoptosis and inhibits proliferation in various cancer cell lines [62]. Notably, these cytotoxic concentrations exhibit substantially lower toxicity toward normal cells, supporting a preferential anticancer effect. This distinction underscores the safety of vanillin-based hydrogels for regenerative applications.

These advanced delivery systems not only protect vanillin from degradation and rapid metabolism but also enable targeted and sustained release, amplifying its pharmacological effects across various applications. By integrating vanillin with polymeric nanoparticles, hydrogels, and other nanocarriers, its therapeutic potential can be fully realized, paving the way for translational research and clinical validation.

Building on these advances, hydrogel-based systems stand out as particularly promising platforms for vanillin delivery due to their tunable physical properties, biocompatibility, and capacity for sustained, localized drug release [63]. Among the various delivery approaches, vanillin-incorporated hydrogels offer a unique combination of therapeutic functionality and material versatility that can be tailored for diverse biomedical applications.

In the following section, we delve deeper into the emerging field of vanillin-based hydrogels, highlighting their synthesis strategies, structural characteristics, and biomedical potential. Particular emphasis will be placed on the roles of vanillin as both a therapeutic agent and a chemical crosslinker or functional moiety in hydrogel networks. We discuss how vanillin’s intrinsic bioactivity can be harnessed or enhanced through hydrogel systems for wound healing, tissue regeneration, cancer therapy, and anti-inflammatory applications. Additionally, we explore the molecular design of vanillin-functionalized polymers and their integration into hydrogel matrices to improve mechanical strength, degradation profiles, and drug-release kinetics.

This focused analysis underscores the transformative potential of vanillin-based hydrogels as next-generation biomaterials for healthcare applications.

## 4. Vanillin-Based Hydrogels: Design, Properties, and Biomedical Applications

### 4.1. Overview of Hydrogels

Hydrogels are three-dimensional, hydrophilic polymer networks capable of retaining substantial amounts of water or biological fluids [64,65,66]. Their soft and elastic nature closely mimics natural tissue, making them highly desirable for various biomedical applications such as wound healing [67], drug delivery [68], tissue engineering [55], and biosensing [69]. The polymeric chains in hydrogels are crosslinked either physically (through hydrogen bonding or ionic interactions) [70,71,72,73] or chemically (via covalent bonds) [74] to form a stable structure that swells but does not dissolve in aqueous environments.

Although vanillin exhibits relatively low aqueous solubility due to its aromatic phenolic structure, several strategies have been established to facilitate its incorporation into hydrogel matrices. These include covalent conjugation of vanillin to hydrophilic polymers [75], encapsulation within nano/microparticles [58] or cyclodextrin complexes [60], solvent-assisted dissolution during crosslinking, and the use of more water-soluble vanillin derivatives such as Schiff-base or methacrylated analogues [8]. These approaches effectively improve vanillin dispersibility and enable its incorporation at higher functional concentrations.

Natural polymers such as chitosan [76], gelatin [77], alginate [78], and hyaluronic acid [79,80] are frequently employed in hydrogel fabrication due to their excellent biocompatibility and biodegradability. Synthetic polymers, including polyethylene glycol (PEG) [81], poly (vinyl alcohol) (PVA) [82], and poly (N-isopropylacrylamide) (PNIPAm) [83], offer tunable mechanical and responsive properties. The integration of bioactive compounds into hydrogels has further advanced their application scope, offering targeted, sustained, and stimuli-responsive therapeutic delivery.

Vanillin, a natural phenolic compound with known antioxidant, antimicrobial, and anti-inflammatory properties, has emerged as a multifunctional additive in hydrogel systems. It can be incorporated as a therapeutic agent or used as a crosslinker due to its reactive aldehyde group. This dual functionality enhances both the structural and biological performance of hydrogels.

Vanillin’s aldehyde group readily forms Schiff-base linkages with primary amines present in polymers such as chitosan, gelatin, or amino-modified hyaluronic acid. These dynamic covalent bonds act as reversible crosslinks that directly influence the gelation rate, mechanical stiffness, viscoelastic behavior, and self-healing properties of the resulting hydrogels. Depending on the design strategy, vanillin can function either as a structural crosslinker or as a pendant bioactive moiety.

The phenolic hydroxyl and methoxy groups of vanillin are responsible for ROS scavenging and antioxidant behavior, enabling hydrogels to modulate oxidative stress during wound healing and tissue regeneration. The aldehyde functionality enhances antibacterial and anticancer effects by promoting protein binding or cellular stress responses. These chemical features, when integrated into hydrogels, significantly contribute to biological outcomes, such as enhanced wound closure, suppression of inflammatory cytokines, and selective cytotoxicity toward cancer cells.

When used as a crosslinker, vanillin increases the crosslinking density and mechanical stability of hydrogels. When attached as a functional side group, vanillin retains its biological activity antioxidant, anti-inflammatory, antibacterial, or anticancer while minimally altering the polymer’s backbone architecture. Building on these material design principles, vanillin-functionalized hydrogels have been explored across a range of biomedical applications where both structural performance and intrinsic bioactivity are required.

### 4.2. Rheological and Mechanical Properties of Vanillin-Based Hydrogels

Rheological behavior plays a critical role in determining the performance of vanillin-based hydrogels. The incorporation of vanillin particularly through dynamic Schiff-base crosslinking often enhances viscoelastic properties by increasing the storage modulus (G’) and crosslinking density. Many vanillin-functionalized hydrogels exhibit pronounced shear-thinning behavior, enabling excellent injectability or printability while rapidly recovering their structure after shear removal [84]. Additionally, vanillin-mediated dynamic covalent bonding contributes to tunable gelation kinetics and self-healing capability, both of which are essential for biomedical applications such as tissue engineering, wound healing, and localized drug delivery.

### 4.3. Vanillin-Based Hydrogels for Wound Healing

Wound healing involves a complex cascade of cellular and molecular events, including inflammation, proliferation, and remodeling [85]. In chronic or infected wounds, prolonged inflammation and microbial colonization delay tissue regeneration [86,87,88]. Hydrogels offer a moist environment that supports healing, while vanillin contributes biological activity.

To harness these benefits, chitosan–vanillin hydrogels have been widely investigated, with vanillin serving dual roles as both an antimicrobial agent and a biocompatible crosslinker. For instance, a recent study investigated a macro-scale chitosan–vanillin (CV) hydrogel as a biocompatible alternative to toxic crosslinkers like glutaraldehyde [89]. Vanillin formed Schiff base linkages and hydrogen bonds with chitosan, producing a highly porous, interconnected 3D hydrogel. Compared to chitosan–glutaraldehyde (CG) hydrogels, the CV hydrogels showed superior porosity (>90%), enhanced swelling behavior, effective antibacterial activity against *E. coli* and *S. aureus*, and good cytocompatibility with L929 and mesenchymal cells. While CV hydrogels exhibited lower mechanical strength in the dry state, they showed improved mechanical properties in the swollen state, highlighting their potential for tissue engineering and wound healing applications.

Building upon these findings, researchers have designed more complex hydrogel systems that incorporate additional functional nanomaterials for enhanced performance. A multifunctional wound dressing was developed using a chitosan-PVA membrane crosslinked with vanillin and reinforced with nanocellulose and CuO-Ag nanoparticles [90]. The membrane showed excellent mechanical strength (49.99 MPa), high moisture retention (98.84%), significant swelling capacity (191.67%), good biodegradability, and high cell viability (92.30%). It also demonstrated strong antimicrobial activity against both Gram-positive and Gram-negative bacteria, along with superior wound healing performance, making it a promising candidate for advanced wound care applications.

Similarly, another study presents a self-gelling hydrogel system composed of chitosan, PVA, and vanillin, designed for enhanced wound healing and skin regeneration [91]. Vanillin acts as a natural crosslinker via Schiff-base reactions with chitosan. The optimized CH–PVA–Van hydrogel offers strong mechanical integrity, self-healing ability, sustained L-arginine release for over 7 days, and high hydrophilicity. It shows potent antimicrobial activity, particularly against Gram-positive bacteria, and promotes rapid wound closure (95% in 24 h) due to nitric oxide release from L-arginine. These features highlight its strong potential as a multifunctional biomaterial for topical wound healing applications.

**Figure 6:**
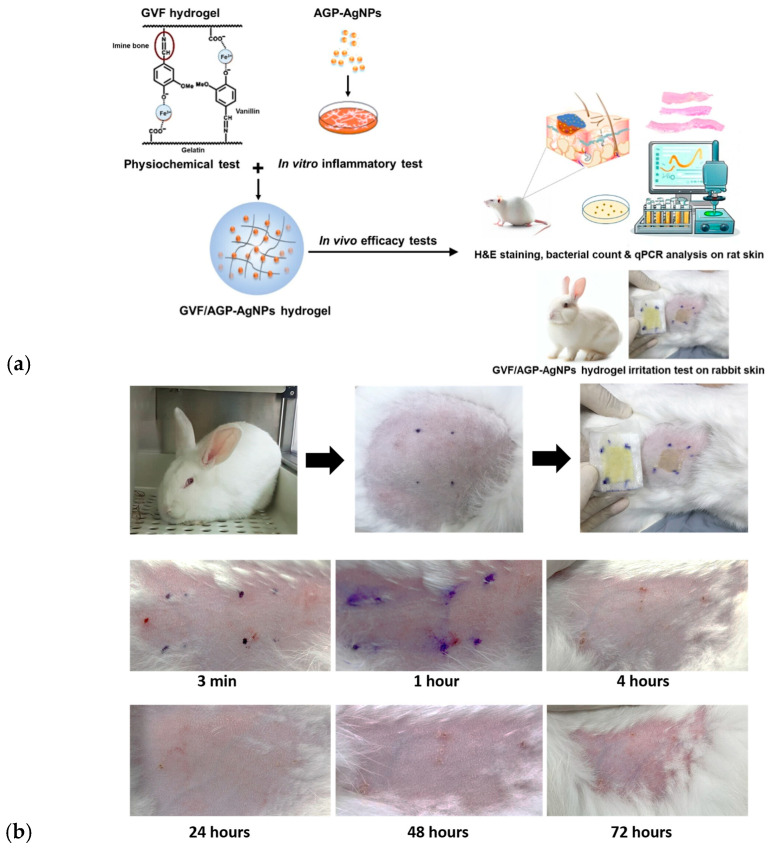
(**a**) Schematic overview of the experimental design and schematic illustrating the evaluation of GVF hydrogels incorporating AGP-AgNPs. The study assessed inflammatory responses using HEKa and RAW 264.7 cell lines and conducted in vivo analyses in rats and rabbits, including histological evaluation, antibacterial testing, and RT-qPCR analysis of rat skin, as well as a dermal irritation test in rabbits. (**b**) Representative image from the rabbit skin irritation test following topical application of GVF/AGP-AgNP hydrogels. Reprinted with permission from Ref. [92]. 2025, American Chemical Society Publications.

In addition to chitosan-based systems, vanillin has also been successfully integrated into gelatin and alginate hydrogels to support wound repair. This study develops a gelatin-based hydrogel (GVF) cross-linked with vanillin and ferric ions, incorporating andrographolide (AGP) and silver nanoparticles (AgNPs), for anti-inflammatory and anti-infective wound healing applications (Figure 6) [92]. The AGP–AgNPs showed low cytotoxicity in human keratinocytes and murine macrophages, while effectively reducing lipopolysaccharide-induced nitric oxide production. In vivo, GVF/AGP–AgNP hydrogels enhanced epidermal regeneration, reduced inflammation, and upregulated key wound-healing genes (e.g., collagen I/III, EGF, TGF-β, VEGF). Additionally, minimal skin irritation was observed in rabbit models. These results support the hydrogel’s potential as a safe and effective topical wound dressing with anti-inflammatory and regenerative capabilities. The incorporation of vanillin not only suppresses microbial activity but also modulates oxidative stress and inflammation, promoting fibroblast proliferation and angiogenesis.

Moreover, recent innovations have focused on combining vanillin with bioadhesive polymers to enable in situ gel formation, which facilitates application to irregular wound surfaces [93,94]. These materials are often thermosensitive or ionically crosslinked, forming a stable gel upon contact with body temperature or physiological fluids.

### 4.4. Vanillin-Based Hydrogels for Anticancer Therapy

**Figure 7:**
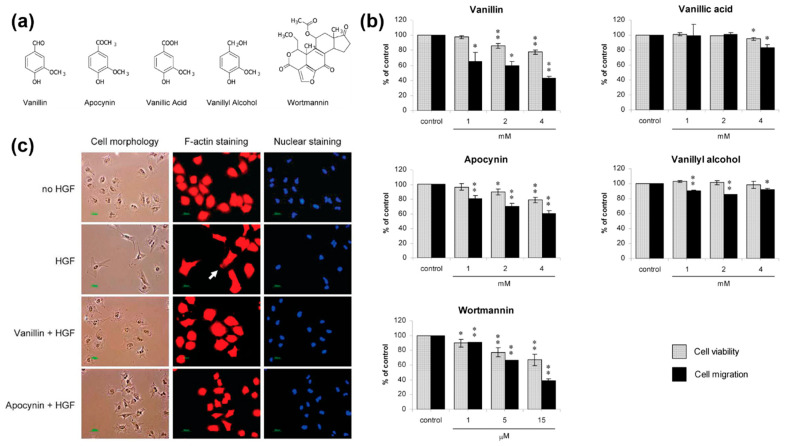
(**a**) Chemical structures of vanillin derivatives and wortmannin (a specific PI3K inhibitor), and their biological effects on HGF-induced A549 lung cancer cell migration and cytoskeletal reorganization. (**b**) Vanillin and its derivatives inhibited HGF-stimulated cell migration and morphological changes in a dose-dependent manner without affecting cell viability. (**c**) Representative fluorescence images show suppression of F-Actin-rich lamellipodia formation after treatment with vanillin or apocynin (scale bar = 50 μm). Reprinted with permission from Ref. [96]. 2017, American Chemical Society Publications. * *p* < 0.05, ** *p* < 0.01.

The application of vanillin in cancer therapy has attracted growing interest due to its demonstrated ability to induce apoptosis, inhibit cell proliferation, and suppress metastasis across various cancer cell lines, including breast, colon, and liver cancers [95]. These anticancer effects are largely attributed to vanillin’s role in modulating oxidative stress, regulating key signaling pathways (such as NF-κB and PI3K/Akt), and influencing the expression of apoptosis-related genes (Figure 7) [96]. However, the therapeutic potential of vanillin is hindered by its poor aqueous solubility, rapid metabolic degradation, and low systemic bioavailability when administered orally or intravenously. To overcome these limitations, hydrogel-based delivery systems have emerged as a promising strategy for localized and sustained drug release.

To address these challenges, recent studies have focused on developing vanillin-based hydrogel nanocomposites, such as vanillin-crosslinked chitosan systems enhanced with ZnO nanoparticles, to improve therapeutic efficacy and controlled drug delivery. Vanillin-crosslinked chitosan (Vn-CS) nanocomposites incorporating various concentrations of ZnO nanoparticles were synthesized and characterized using FTIR, XRD, TGA, SEM, and TEM [97]. Enhanced antibacterial activity and 5-fluorouracil (5-FU) encapsulation efficiency (61.4–69.2%) were observed with increasing ZnO content. Drug release was monitored via a mesoporous ZrO_2_–Co_3_O_4_-modified sensor and analyzed using kinetic models. Cytotoxicity assays revealed that 5-FU-loaded Vn-CS nanocomposites, particularly 5-FU/CV10, showed strong antitumor effects against HePG-2, MCF-7, and HCT-116 cells, with minimal toxicity to normal WI-38 and WISH cells. These findings highlight their potential as selective, low-toxicity anticancer nanocarriers.

### 4.5. Vanillin-Based Hydrogels for Anti-Inflammatory Applications

Inflammation is a key underlying factor in many chronic conditions such as arthritis, dermatitis, and autoimmune diseases, often contributing to tissue damage and disease progression. Vanillin, a naturally occurring phenolic compound, has been shown to possess potent anti-inflammatory properties by inhibiting the production of major pro-inflammatory cytokines including IL-1β, IL-6, and TNF-α [98]. This inhibition is primarily achieved through the modulation of the NF-κBsignaling pathway, which plays a crucial role in regulating immune and inflammatory responses [99]. However, the therapeutic efficacy of vanillin is often limited by its rapid metabolism and poor bioavailability. To overcome these challenges, encapsulating vanillin within hydrogel matrices provides a promising approach, enabling localized and sustained release of the compound directly at inflamed tissues. This targeted delivery not only enhances vanillin’s anti-inflammatory effects but also reduces systemic side effects, improving its potential for clinical applications in managing chronic inflammatory diseases.

Building on vanillin’s anti-inflammatory potential, recent research has explored its integration with biomaterials to create advanced therapeutic platforms. One such approach involves combining vanillin with biocompatible hydrogels to not only deliver the compound effectively but also support tissue regeneration, particularly in degenerative diseases. This study presents a smart injectable hydrogel composed of vanillin and hyaluronic acid, crosslinked to encapsulate intervertebral disk-derived stem cells (IVDSCs) for treating nucleus pulposus (NP) degeneration [100]. The hydrogel demonstrated improved mechanical strength, shear-thinning behavior, and maintained cell viability up to 500 µg/mL. It supported IVDSC proliferation, multilineage differentiation, and reduced inflammatory cytokine release. Gene expression indicated enhanced chondrocyte marker levels. In vivo injection led to increased disk height, better histological outcomes, and improved biomechanical properties, suggesting the hydrogel as a promising carrier for IVD regeneration therapy.

In addition to hydrogel-based stem cell delivery systems, another promising strategy leverages vanillin’s multifunctionality through chemical functionalization of biomaterial carriers. For example, this work presents a vanillin-functionalized gelatin methacrylate (GelMA) microsphere system engineered for the localized and sustained delivery of transforming growth factor-β3 (TGF-β3) to target intervertebral disk degeneration (IVDD) [101]. The incorporation of vanillin, a phenolic aldehyde with inherent antioxidant and anti-inflammatory properties, into the GelMA matrix enhances the biofunctionality of the microspheres. In vitro assays demonstrated improved TGF-β3 release kinetics, downregulation of pro-inflammatory cytokines, and upregulated extracellular matrix (ECM) production in lipopolysaccharide (LPS)-stimulated nucleus pulposus (NP) cells. In vivo studies confirmed that the system mitigates oxidative stress and inflammation, preserves disk hydration and height, and maintains biomechanical integrity post-treatment. Transcriptomic profiling revealed that suppression of the PI3K/Akt signaling pathway may underpin the therapeutic effect. These findings establish vanillin-mediated chemical functionalization as a robust strategy to confer multifunctionality to biomaterial carriers for regenerative therapies in IVDD and potentially other degenerative tissue disorders.

Similarly, vanillin’s versatile bioactivity has been harnessed in wound healing applications. By formulating composite hydrogels with polymers like polyvinyl alcohol and chitosan, researchers have developed multifunctional dressings that combine mechanical resilience with antimicrobial and antioxidant properties. This study developed a three-phase hydrogel dressing composed of polyvinyl alcohol (PVA), chitosan (CS), and vanillin (V) via freeze–thaw cycles to enhance wound healing [93]. Compared to PVA/V and PVA/CS hydrogels, the PVA/CS/V (PCV) hydrogel maintained an elastic modulus above 5 kPa across 15–40 °C and demonstrated superior antibacterial activity against both Gram-positive and Gram-negative bacteria. It also showed strong antioxidant effects by effectively scavenging various free radicals (DPPH, ABTS+, PTIO). The PCV hydrogel’s combination of simple preparation and excellent bioactivity indicates strong potential for commercial wound dressing applications.

To further expand the versatility of vanillin-functionalized biomaterials, recent efforts have introduced vanillin into gelatin-based hydrogel platforms, enabling superior mechanical integrity and antibacterial performance suited for regenerative applications. Rao et al. (2025) synthesized a gelatin methacryloyl–vanillin (GelMa-V) hydrogel using a visible-light photochemical approach with Eosin-Y as the initiator and triethanolamine/N-vinyl caprolactam as co-initiators [102]. The incorporation of vanillin enhanced gelation kinetics and structural integrity through Schiff-base bond formation with GelMa. Structural analyses (FTIR, XRD, SEM) revealed a transition from crystalline to amorphous architecture and an interconnected porous morphology favorable for nutrient diffusion and cell attachment. Mechanical testing demonstrated that vanillin increased the hydrogel’s compressive strength, swelling capacity, and tissue adhesion, while release studies confirmed a controlled vanillin release profile. The GelMa-V hydrogel also showed excellent biocompatibility with NIH-3T3 fibroblasts and strong antibacterial activity against E. coli (up to 98% inhibition). Overall, GelMa-V represents a multifunctional and tunable hydrogel combining mechanical robustness, antibacterial efficacy, and cytocompatibility, underscoring its potential for wound healing and regenerative medicine applications.

### 4.6. Vanillin as a Crosslinker or Functional Moiety in Hydrogel Networks

**Scheme 1:**
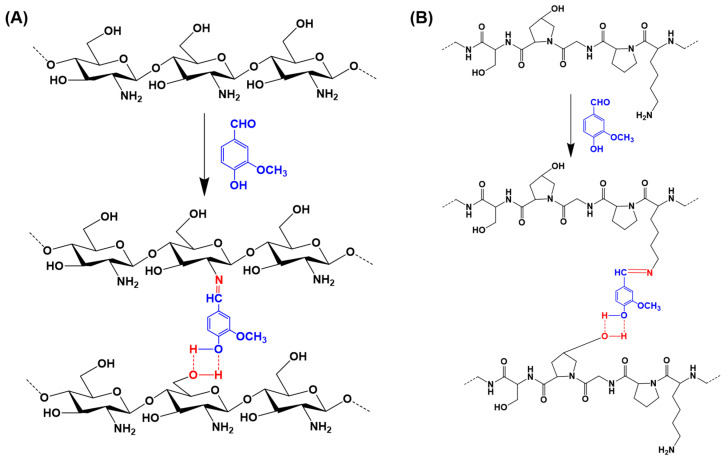
Synthesis scheme illustrating the chemical bonding of vanillin within the (**A**) chitosan and (**B**) Gelatin hydrogel network.

Beyond its well-recognized therapeutic properties, vanillin’s chemical structure, particularly its reactive aldehyde group, enables it to form covalent bonds with polymers containing amine groups through the formation of reversible imine (Schiff base) linkages (Scheme 1) [103]. This unique chemical reactivity has been effectively exploited to engineer advanced hydrogel systems that are self-healing, injectable, and responsive to environmental stimuli such as pH or temperature changes. In addition to its role in structural crosslinking, vanillin also imparts intrinsic antioxidant and antimicrobial properties to hydrogel matrices, thereby improving their functional performance in biological environments. The phenolic hydroxyl group of vanillin can scavenge free radicals and inhibit bacterial growth, which contributes to prolonged material stability and enhanced tissue compatibility in vivo.

In self-healing hydrogels, vanillin serves as a dynamic crosslinker by providing reversible covalent bonds that can break and reform under mechanical stress or damage [104]. This dynamic bonding capacity allows the hydrogel to restore its integrity and mechanical properties autonomously after deformation or rupture, making such materials particularly attractive for biomedical implants, wound dressings, and tissue scaffolds that are subjected to frequent movement or mechanical disturbance in vivo. Recent studies have demonstrated that combining vanillin with dual crosslinking mechanisms such as Schiff base formation and photopolymerization can further enhance the balance between mechanical strength and dynamic self-repairing behavior, providing adaptable materials suitable for minimally invasive biomedical applications.

Vanillin-crosslinked hydrogels based on natural polymers such as gelatin and chitosan have demonstrated tunable physicochemical properties [89,105]. By varying the molar ratio of vanillin to polymer amine groups, researchers can precisely control the crosslinking density, which directly impacts the hydrogel’s mechanical strength, swelling capacity, degradation rate, and drug release profiles. This tunability allows for customization of the hydrogel characteristics to suit specific biomedical applications, ranging from sustained drug delivery to scaffold-based tissue engineering. Notably, vanillin’s dual functionality—as both a structural crosslinker and bioactive additive enables simultaneous mechanical reinforcement and therapeutic modulation, bridging material design with biological performance in a single system.

Moreover, vanillin-based crosslinked networks can be further functionalized by integrating additional components such as magnetic nanoparticles [106], photosensitizers [107], or bioactive peptides that promote cell adhesion [108]. These multifunctional hydrogels open new avenues for advanced biomedical therapies, including magnetically guided tissue regeneration, photodynamic cancer treatment, and the development of highly biointeractive scaffolds that mimic native extracellular matrices. Emerging research also suggests that vanillin-containing hybrid hydrogels can serve as controlled-release depots for antioxidants or antibiotics, enabling localized and sustained therapeutic delivery while simultaneously supporting tissue regeneration.

### 4.7. Future Perspectives

The integration of vanillin into hydrogel systems has opened promising avenues in the field of biomedical materials, combining the structural versatility of hydrogels with the pharmacological potency of vanillin [109]. While substantial progress has been made in demonstrating their therapeutic benefits in wound healing [110], cancer therapy [111], antimicrobial coatings [112], and tissue engineering [105], several important directions remain underexplored. Rather than reiterating established applications, this section highlights unresolved translational barriers and emerging design strategies that define the next phase of vanillin-based hydrogel research. Future research must strategically address these gaps to fully unlock the clinical and translational potential of vanillin-based hydrogels.

Despite extensive preclinical evaluation, most vanillin-based hydrogels have not progressed beyond the laboratory or early animal studies [101]. One key challenge is regulatory approval. For clinical translation, comprehensive toxicological profiling [113], biodegradation studies [114], and long-term biocompatibility [115] assessments are essential. Moreover, Good Manufacturing Practice (GMP)-compliant synthesis routes for vanillin-functionalized polymers or injectable hydrogels must be developed. Establishing reproducible fabrication protocols and scalable manufacturing technologies will be critical to facilitate clinical trials and commercial development.

Another promising direction is the development of stimuli-responsive and multifunctional vanillin-based hydrogels. Smart hydrogels that respond to environmental triggers such as pH [116], temperature [117], redox potential [118], or enzymatic activity [74] could offer site-specific and on-demand drug delivery capabilities. For instance, designing thermo- or pH-sensitive hydrogels for targeted anticancer therapy or inflammation-responsive systems for chronic wounds can enhance the therapeutic index of vanillin [119]. Moreover, incorporating dual or multi-drug systems with synergistic bioactives [120], such as antibiotics, growth factors, or anti-inflammatory agents, into vanillin-based matrices can yield next-generation hydrogels for complex clinical scenarios.

Current studies have largely focused on commonly used natural polymers such as chitosan, alginate, and hyaluronic acid [121]. Future research should explore emerging biopolymers, including silk fibroin [122], gelatin methacryloyl [123], bacterial cellulose [124], and peptide-based hydrogels [77], as potential backbones for vanillin incorporation. In addition, green and bio-orthogonal crosslinking techniques such as click chemistry or enzyme-mediated crosslinking can minimize toxicity and allow precise structural control [125]. Vanillin’s aldehyde group offers a chemically active handle for diverse crosslinking reactions, enabling customized hydrogel architectures and mechanical properties.

There is growing interest in utilizing vanillin-based hydrogels in regenerative medicine, particularly for bone, cartilage, and neural tissue engineering [89]. Vanillin’s anti-inflammatory and antioxidant properties are especially attractive for modulating the immune environment and enhancing tissue integration [126]. Further studies are needed to evaluate cell–material interactions, angiogenesis potential, and in vivo degradation behavior in large animal models. Personalized therapy is another emerging application. Three-dimensional printing and bioprinting technologies can be leveraged to fabricate patient-specific vanillin-containing hydrogel constructs with tailored mechanical and biochemical cues [127].

Combining vanillin with other natural or synthetic bioactive materials may unlock synergistic therapeutic effects. For example, integrating vanillin with curcumin [128], resveratrol [129], or growth factors could enhance antioxidant [130], anti-inflammatory, and regenerative responses in complex wound or cancer environments [131]. Furthermore, co-loading vanillin with chemotherapeutics or immunomodulators in hydrogel systems can improve efficacy while reducing systemic toxicity, especially in localized treatment of solid tumors or chronic inflammatory disorders [91,132].

Given increasing global emphasis on sustainability and biowaste valorization, vanillin’s derivation from lignin and agro-industrial residues positions it as a valuable component of the circular bioeconomy [133]. Future hydrogel design strategies should consider the eco-friendly sourcing and lifecycle of both vanillin and the supporting polymer matrix [134]. Development of biodegradable, low-carbon-footprint hydrogel systems aligns with global trends toward sustainable healthcare materials.

## 5. Conclusions

Vanillin, traditionally recognized as a flavoring agent, has emerged as a bioactive compound with significant pharmacological potential. Its antioxidant and anti-inflammatory properties play a critical role in mitigating oxidative stress and inflammation-related diseases, while its anticancer effects are attributed to its ability to suppress tumor progression, induce apoptosis, and inhibit key signaling pathways such as NF-κB and MAPK. Additionally, vanillin’s antimicrobial activity highlights its value as both a food preservative and a therapeutic agent against bacterial and fungal pathogens. Despite promising preclinical evidence, challenges such as low solubility, limited bioavailability, and a lack of robust clinical trials hinder its broader therapeutic application. Notably, the current literature lacks a unified framework linking vanillin’s chemical functionality, hydrogel network design, and resulting biological performance, particularly in the context of clinical translation.

To fully realize vanillin’s potential, several future directions must be pursued. Rigorous clinical trials are needed to validate its safety, efficacy, and therapeutic dosage across various health conditions, including cancer, inflammatory diseases, and neurodegenerative disorders. Overcoming bioavailability limitations through advanced drug delivery systems, such as nanoparticles and targeted formulations, will enhance its systemic absorption and therapeutic performance. Structure–activity relationship studies and multi-omics approaches, including genomics, proteomics, and metabolomics, could provide deeper insights into vanillin’s molecular mechanisms and optimize its bioactivity. Moreover, exploring its integration into functional foods and nutraceuticals could bridge its therapeutic benefits with preventive healthcare strategies. Importantly, this review identifies critical knowledge gaps including concentration-dependent cytotoxicity, long-term biocompatibility, pharmacokinetics, and hydrogel–tissue interactions that currently limit rational design and clinical translation. Addressing these gaps will guide the rational design of next-generation vanillin-based hydrogels with enhanced therapeutic performance.

In addition, vanillin’s antimicrobial properties warrant further investigation to combat antibiotic-resistant pathogens, addressing global health challenges. Sustainable production methods, such as biotechnological approaches for plant-derived or microbial synthesis, will be crucial to ensure scalability and accessibility. By addressing these challenges and opportunities, vanillin holds substantial promise as a multifunctional compound capable of contributing to therapies in oncology, food safety, and chronic disease management while enhancing human health and well-being.

Looking ahead, several promising research directions remain unexplored. Future studies should evaluate long-term biocompatibility, degradation behavior, and in vivo pharmacokinetics of vanillin-based hydrogels. The optimization of vanillin concentration, dynamic covalent crosslinking chemistry, and multifunctional hydrogel architectures also presents important opportunities. Moreover, integrating vanillin with advanced biomaterial systems such as conductive hydrogels, 3D-printed scaffolds, and stimuli-responsive networks may further expand its therapeutic potential. By consolidating structure–function relationships, delivery strategies, and translational challenges, this review provides a focused framework to guide the rational development of next-generation vanillin-based hydrogels for biomedical applications.

## Data Availability

The original contributions presented in this study are included in the article. Further inquiries can be directed to the corresponding authors.
